# New York City House Mice (Mus musculus) as Potential Reservoirs for Pathogenic Bacteria and Antimicrobial Resistance Determinants

**DOI:** 10.1128/mBio.00624-18

**Published:** 2018-04-17

**Authors:** Simon H. Williams, Xiaoyu Che, Ashley Paulick, Cheng Guo, Bohyun Lee, Dorothy Muller, Anne-Catrin Uhlemann, Franklin D. Lowy, Robert M. Corrigan, W. Ian Lipkin

**Affiliations:** a Center for Infection and Immunity, Columbia University, New York, New York, USA; b Division of Healthcare Quality Promotion, Centers for Disease Control and Prevention, Atlanta, Georgia, USA; c Division of Infectious Diseases, Department of Medicine, Columbia University, New York, New York, USA; d RMC Pest Management Consulting, Briarcliff Manor, New York, USA; University of Maryland, School of Medicine

**Keywords:** antimicrobial resistance, bacteriome, mice, New York City

## Abstract

House mice (Mus musculus) thrive in large urban centers worldwide. Nonetheless, little is known about the role that they may play in contributing to environmental contamination with potentially pathogenic bacteria. Here, we describe the fecal microbiome of house mice with emphasis on detection of pathogenic bacteria and antimicrobial resistance genes by molecular methods. Four hundred sixteen mice were collected from predominantly residential buildings in seven sites across New York City over a period of 13 months. 16S rRNA sequencing identified *Bacteroidetes* as dominant and revealed high levels of *Proteobacteria*. A targeted PCR screen of 11 bacteria, as indicated by 16S rRNA analyses, found that mice are carriers of several gastrointestinal disease-causing agents, including *Shigella*, *Salmonella*, Clostridium difficile, and diarrheagenic Escherichia coli. Furthermore, genes mediating antimicrobial resistance to fluoroquinolones (*qnrB*) and β-lactam drugs (*bla*_SHV_ and *bla*_ACT/MIR_) were widely distributed. Culture and molecular strain typing of C. difficile revealed that mice harbor ribotypes associated with human disease, and screening of kidney samples demonstrated genetic evidence of pathogenic *Leptospira* species. In concert, these findings support the need for further research into the role of house mice as potential reservoirs for human pathogens and antimicrobial resistance in the built environment.

## IMPORTANCE

Mice are commensal pests often found in close proximity to humans, especially in urban centers. We surveyed mice from seven sites across New York City and found multiple pathogenic bacteria associated with febrile and gastrointestinal disease as well as an array of antimicrobial resistance genes.

## INTRODUCTION

The house mouse (Mus musculus) commonly lives indoors with humans, inhabits all continents except Antarctica (1), and has been found to carry pathogenic microbes, including Clostridium difficile (2), *Campylobacter* (3), *Leptospira* spp. (4–6), Toxoplasma gondii (7), and *Salmonella* (8). Wild house mice in New York City (NYC) have been linked to an outbreak of rickettsialpox (9). A survey of M. musculus obtained from pet shops indicated carriage of Enterococcus faecium (10).

Antimicrobial resistance (AMR) poses an important threat to human health. Several reports describe immediate concerns for NYC, including increasing rates of resistance in *Shigella* spp., multidrug-resistant Klebsiella pneumoniae, and the emergence of colistin resistance (11–13). AMR in both pathogenic and nonpathogenic bacteria is commonly mediated by mobile genetic elements (14). Rodents have been shown to be carriers of antibiotic-resistant bacteria (15–17). To our knowledge, there are no published surveys of the house mouse microbiome and resistome in large urban centers such as NYC.

Mice are widely distributed in NYC across geographic regions and economic strata (18). Given their close proximity to humans and their potential to contaminate their local environment with pathogenic organisms, we trapped wild house mice inside multiunit residential buildings in NYC and surveyed them for the presence of pathogenic bacteria and AMR genes. We applied a two-tiered discovery approach using bacterial 16S rRNA sequencing and commercial multiplexed AMR PCR arrays on pooled fecal samples followed by targeted PCR of individual anal swabs. We also directly screened mouse kidneys for the presence of *Leptospira* DNA because this bacterium concentrates in the urinary tract.

## RESULTS

### Mouse trapping and sample collection.

**FIG 1:**
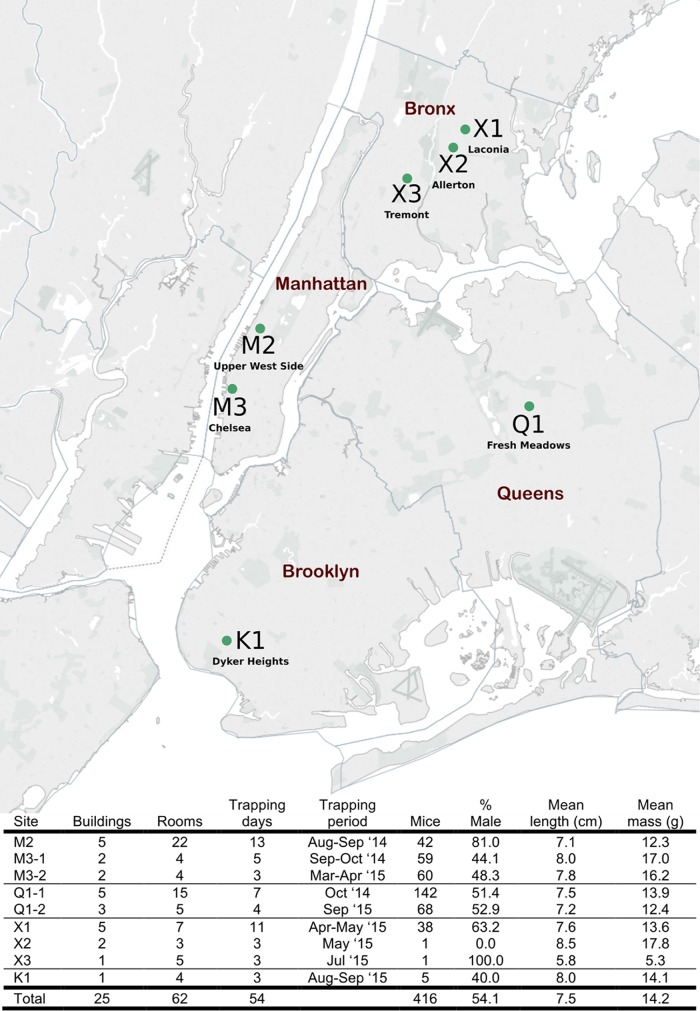
Site locations in New York City and house mouse population summary. Map created with Tableau Software and published with permission of the company.

The population of mice used in this study was also used in a parallel study that examined the virome of house mice in NYC (Fig. 1) (19). Both single and multicatch traps were used. Multicatch traps included from 1 to 11 mice. Where traps included more than one mouse, fecal pellets were pooled. Fecal pellets were used for 16S rRNA sequencing and AMR surveys. Anal swabs from individual mice were used for confirmatory PCR assays and bacterial culture. Kidneys were used for *Leptospira* surveys.

### Fecal 16S rRNA sequencing.

**FIG 2:**
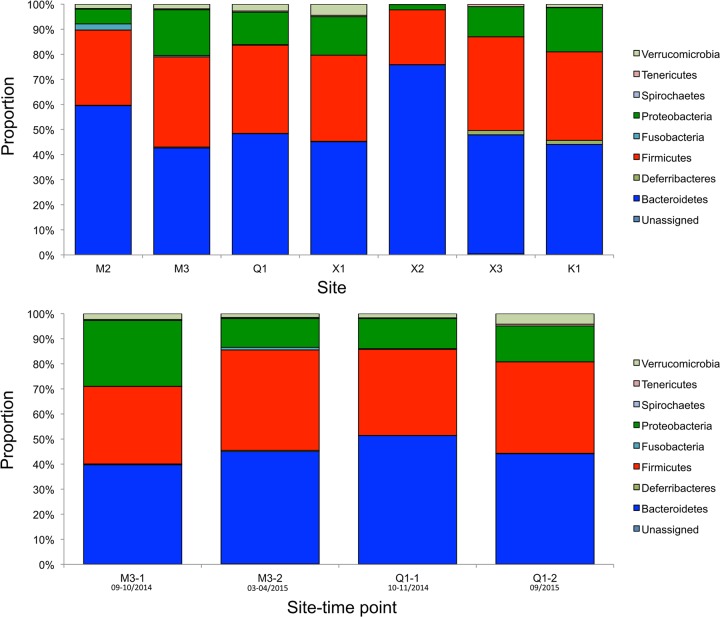
Bacterial phyla of wild house mice in New York City. Histograms represent the average proportion of operational taxonomic units that were assigned to bacterial phyla from all samples from the same site or time point. (Top) Comparison of the fecal microbiomes from seven sites. (Bottom) Variation in fecal microbiome compositions between two time points at two sites. Month/year of collection is provided under each site-time point.

An average of 116,334 quality-filtered reads were generated per sample from 107 pooled or individual fecal pellet samples; three samples produced fewer than 329 reads and were excluded. The fecal bacterial population structures at each site were consistent at the phylum level. Sequencing of the bacterial 16S rRNA V4 region identified *Bacteroidetes* as the most dominant population at each site (range, 42% to 76%), followed by *Firmicutes* (22% to 37%) and *Proteobacteria* (2% to 18%) (Fig. 2, top; see Table S1 in the supplemental material). Comparison at two sites (located 16.35 km apart) over two collection points indicated that the fecal microbiota of wild house mice in NYC were stable at the phylum level, with *Bacteroidetes* remaining the most abundant, followed by *Firmicutes* and *Proteobacteria* (Fig. 2, bottom). Based on the available bacterial 16S rRNA V4 sequences, 235 genera and 149 species were taxonomically defined.

10.1128/mBio.00624-18.1 TABLE S1  (A) Microbiome composition determined from 16S rRNA V4 sequencing of pooled fecal pellets from NYC house mice at seven unique sites. Values represent average proportions of operational taxonomic units. (B) Microbiome composition determined from 16S rRNA V4 sequencing of pooled fecal pellets from NYC house mice at two sites, each with two collection time points. Values represent average proportions of operational taxonomic units assigned to each phylum. Download TABLE S1, DOCX file, 0.1 MB. Copyright © 2018 Williams et al. 2018 Williams et al. This content is distributed under the terms of the Creative Commons Attribution 4.0 International license.

### PCR screens for pathogenic bacteria.

Individual anal swabs were tested by PCR to determine the prevalence of bacteria found in 16S rRNA analyses of pooled fecal samples. We used established assays for detection of C. difficile (*tcdB*) (20), *Salmonella* (*invA*) (21), enterotoxigenic *Escherichia coli* (*st*) (22), typical and atypical enteropathogenic E. coli (EPEC) (*eae*) (23), enteroaggregative E. coli (EAEC) (*aggR*) (24), Shiga toxin-producing E. coli (STEC) (*stx*_1_ and *stx*_2_) (25), and K. pneumoniae (16S rRNA and *khe*) (26, 27) and three new assays specifically designed and validated for this study to detect enteroinvasive E. coli (EIEC)/*Shigella* (*ipaH*), typical EPEC (*bfpA*), and Clostridium perfringens (*cpa*) (Table S2). C. perfringens, atypical EPEC (*eae*^+^/*bfpA*^−^), and K. pneumoniae were detected in four sites (M2, M3, Q1, and X1) distributed across three boroughs (Table 1). Bacteria associated with gastrointestinal disease in humans were detected in this study, including *Shigella*/EIEC (60/416, 14%), C. perfringens (48/416, 12%), atypical EPEC (18/416, 4%), C. difficile (18/416, 4%), and *Salmonella* spp. (13/416, 3%) (Table 1).

10.1128/mBio.00624-18.2 TABLE S2  Typed strains used for PCR assay specificity testing. Download TABLE S2, DOCX file, 0.1 MB. Copyright © 2018 Williams et al. 2018 Williams et al. This content is distributed under the terms of the Creative Commons Attribution 4.0 International license.

**TABLE 1:** PCR detection of bacterial targets in individual mice^a^

Bacterium	Target gene	% prevalence by PCR at site:							% of positive mice by:					Total (%)
									Sex		Age			
		M2	M3	Q1	X1	X2	X3	K1	M	F	J	S	A	
C. difficile	*tcdB*	7/42 (16.7)	2/119 (1.7)	9/210 (4.3)	0/38 (0.0)	0/1 (0.0)	0/1 (0.0)	0/5 (0.0)	8/18 (44)	10/18 (56)	13/18 (72)	0/18 (0)	5/18 (28)	18/416 (4.3)
C. perfringens	*cpa*	8/42 (19.0)	14/119 (11.8)	25/210 (11.9)	1/38 (2.6)	0/1 (0.0)	0/1 (0.0)	0/5 (0.0)	24/48 (50)	24/48 (50)	18/48 (38)	5/48 (10)	25/48 (52)	48/416 (11.5)
*Salmonella* spp.	*invA*	2/42 (4.8)	2/119 (1.7)	9/210 (4.3)	0/38 (0.0)	0/1 (0.0)	0/1 (0.0)	0/5 (0.0)	6/13 (46)	7/13 (54)	7/13 (54)	2/13 (15)	4/13 (31)	13/416 (3.1)
*Shigella*/EIEC	*ipaH*	4/42 (9.5)	23/119 (19.3)	33/210 (15.7)	0/38 (0.0)	0/1 (0.0)	0/1 (0.0)	0/5 (0.0)	33/60 (55)	27/60 (45)	30/60 (50)	12/60 (20)	18/60 (30)	60/416 (14.4)
ETEC	*st*	0/42 (0.0)	0/119 (0.0)	0/210 (0.0)	0/38 (0.0)	0/1 (0.0)	0/1 (0.0)	0/5 (0.0)	0/0 (0)	0/0 (0)	0/0 (0)	0/0 (0)	0/0 (0)	0/416 (0.0)
EPEC (a/t)	*eae*	2/42 (4.8)	3/119 (2.5)	12/210 (5.7)	1/38 (2.6)	0/1 (0.0)	0/1 (0.0)	0/5 (0.0)	12/18 (67)	6/18 (33)	9/18 (50)	3/18 (17)	6/18 (33)	18/416 (4.3)
EPEC (t)	*bfpA*	0/42 (0.0)	0/119 (0.0)	0/210 (0.0)	0/38 (0.0)	0/1 (0.0)	0/1 (0.0)	0/5 (0.0)	0/0 (0)	0/0 (0)	0/0 (0)	0/0 (0)	0/0 (0)	0/416 (0.0)
EAEC	*aggR*	0/42 (0.0)	5/119 (4.2)	0/210 (0.0)	0/38 (0.0)	0/1 (0.0)	0/1 (0.0)	0/5 (0.0)	2/5 (40)	3/5 (60)	1/5 (20)	0/5 (0)	4/5 (80)	5/416 (1.2)
STEC	*stx*_1_	0/42 (0.0)	0/119 (0.0)	0/210 (0.0)	0/38 (0.0)	0/1 (0.0)	0/1 (0.0)	0/5 (0.0)	0/0 (0)	0/0 (0)	0/0 (0)	0/0 (0)	0/0 (0)	0/416 (0.0)
STEC	*stx*_2_	0/42 (0.0)	1/119 (0.8)	0/210 (0.0)	0/38 (0.0)	0/1 (0.0)	0/1 (0.0)	0/5 (0.0)	1/1 (100)	0/1 (0)	1/1 (100)	0/1 (0)	0/1 (0)	1/416 (0.2)
K. pneumoniae	16S rRNA/*khe*	4/42 (9.5)	6/119 (5.0)	25/210 (11.9)	4/38 (10.5)	0/1 (0.0)	0/1 (0.0)	0/5 (0.0)	21/39 (54)	18/39 (46)	8/39 (20)	10/39 (26)	21/39 (54)	39/416 (9.4)
*Leptospira* spp.	16S rRNA	2/42 (4.8)	6/119 (5.0)	4/172 (2.3)	2/38 (5.3)	0/1 (0.0)	0/1 (0.0)	0/5 (0.0)	11/14 (79)	3/14 (21)	3/14 (21)	2/14 (14)	9/14 (64)	14/378 (3.7)

a Abbreviations: A, adult; a, atypical; EAEC, enteroaggregative *E. coli*; EIEC, enteroinvasive *E. coli*; EPEC, enteropathogenic *E. coli*; ETEC, enterotoxigenic *E. coli*; F, female; J, juvenile; M, male; SA, subadult; STEC, Shiga toxin-producing *E. coli*; t, typical.

### PCR screen for *Leptospira* spp. and phylogenetic analysis.

*Leptospira* DNA was detected by PCR in the kidney tissue of 14/378 (4%) mice. Sequencing of the 291-nucleotide (nt) PCR product indicated the presence of two genotypes most closely related to Leptospira interrogans or Leptospira kirschneri. Sequence analysis of an extended region of the 16S rRNA gene (1,198 nt) revealed the presence of two genotypes that were 98.8% identical to each other and 100% identical within each group. Sequences from group 1 (*n* = 8) were widely distributed in Manhattan, Queens, and the Bronx and were 99.50% identical to both L. kirschneri and L. interrogans. The group 2 sequences (*n* = 4), restricted to both sites in Manhattan, were 99.42% identical to L. kirschneri and 99.33% identical to L. interrogans.

**FIG 3:**
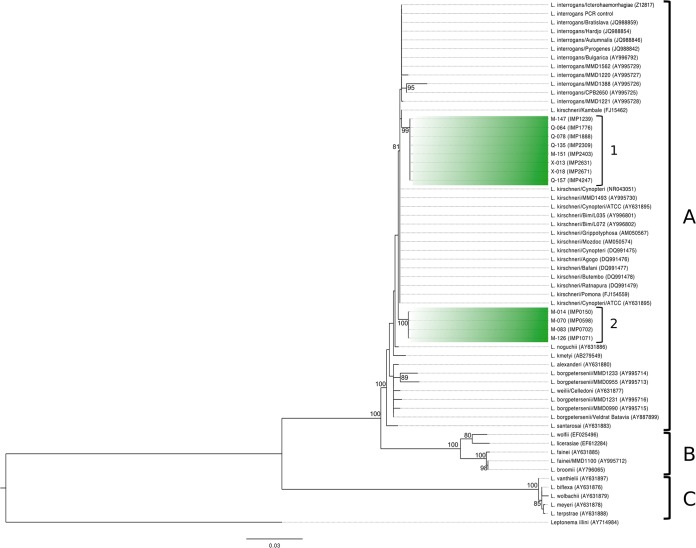
Maximum likelihood tree of the 16S rRNA gene for selected *Leptospira* species with Leptonema illini as the outgroup. The scale bar represents units of substitutions per site. Sequences identified in this study are indicated as either group 1 or group 2. Group A, pathogenic strains; group B, intermediate strains; group C, saprophytic strains. Bootstrap nodal support values are indicated if >70.

Phylogenetic analysis of the near-complete 16S rRNA gene confirmed that all *Leptospira* sequences clustered into the larger complex of pathogenic strains (“A”) (28) (Fig. 3). Group 1 strains clustered with L. interrogans and L. kirschneri, while group 2 formed a separate clade from known L. interrogans and L. kirschneri 16S rRNA sequences.

### C. difficile culture and molecular characterization.

Cytotoxin B (*tcdB*) DNA was detected by PCR in the anal swabs of 18 mice from sites M2, M3-2, Q1-1, and Q1-2. Bacterial culture was then performed using the fecal pellet from a subset of these mice to obtain a representative isolate from each trapping site. Culture was successful for five samples from a total of eight samples where culture was attempted (62.5% success rate). One colony from each plate was selected for further characterization. Two isolates (Q1-1 [2377] and Q1-2 [4697]) were obtained from pooled fecal pellets (i.e., from multiple mice). Therefore, it is not possible to determine which individual mouse was the source of these particular isolates. The remaining three isolates were obtained from M2 (0098 and 0298) and M3-2 (1147) fecal pellets sourced from individual mice. All isolates were positive for both cytotoxin genes (*tcdA/tcdB*) and negative for the binary toxin genes (*cdtA*/*cdtB*) (Table 2). No deletions were found within the *tcdC* target region. Three unique ribotypes (RTs) were identified from the 5 isolates, with each RT associated with a separate site (M2, RT021; M3, RT106; Q1, RT005). RT005 was identified in isolates from samples collected at both time points from site Q1 (i.e., Q1-1 and Q1-2 [Table 2]). The Emerging Infections Program (EIP) for C. difficile infections at the Centers for Disease Control and Prevention has isolated each of these RTs from human cases (29).

**TABLE 2:** Characterization of Clostridium difficile isolates^a^

Site	Isolate ID	Source	Ribotype	Non-tox	*tcdA*	*tcdB*	*cdtA*	*cdtB*	*tcdC* deletion
M2	0098-1.1	FP	21	−	+	+	−	−	ND
M2	0298-2.1	FP	21	−	+	+	−	−	ND
M3-2	1147-2.1	FP	106	−	+	+	−	−	ND
Q1-1	2377-4	Pooled FP	5	−	+	+	−	−	ND
Q1-2	4697-4	Pooled FP	5	−	+	+	−	−	ND

a Abbreviations: ID, identifier; FP, fecal pellet; ND, not detected; Non-tox, nontoxigenic.

### Characterization of AMR determinants by PCR.

**FIG 4:**
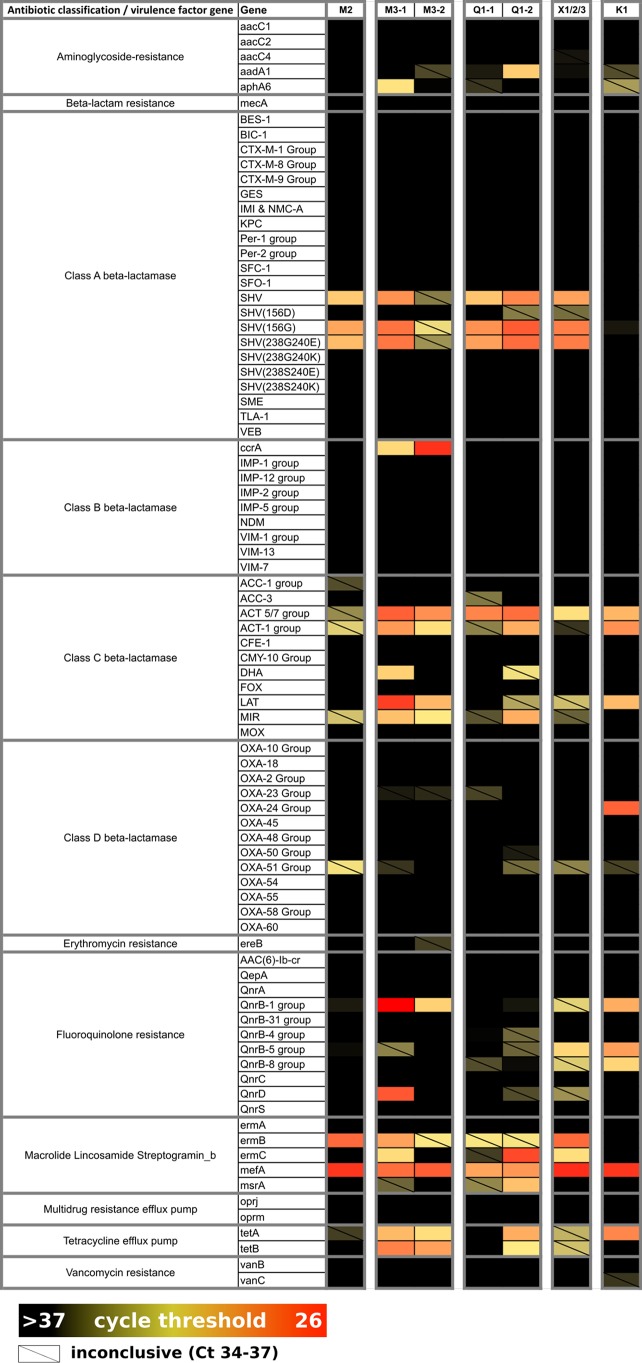
Antimicrobial resistance profiles from house mouse fecal contents in New York City. Cells are colored according to the intensity of the qPCR cycle threshold, where a lower value (red) indicates a higher concentration of the target gene, relative to other samples for the same assay. All *C*_*T*_ values of >37 were considered negative (black), and *C*_*T*_ values between 34 and 37 were recorded as inconclusive (diagonal slash).

Quantitative PCR (qPCR) screening of pooled and individual fecal pellet samples using the 84-AMR-target microbial DNA qPCR array (Qiagen, Valencia, CA) revealed the presence of 22 AMR targets. Genes encoding quinolone resistance (*qnrB* and *qnrD*); macrolide resistance (*mefA*, *ermB*, and *ermC*); and class A (*bla*_SHV_), class B (*ccrA*), class C (*bla*_ACT_ 5/7 and *LAT*), and class D (OXA-24) β-lactamases were each detected with a cycle threshold (*C*_*T*_) of <30 and represent the most strongly reactive targets (Fig. 4). We did not detect *mecA* by qPCR.

For enhanced resolution for three of these AMR genes (*bla*_ACT_, *qnrB*, and *bla*_SHV_) and to further interrogate *mecA* prevalence, we screened all anal swabs (*n* = 416) by PCR using established assays for detection of *bla*_SHV_ (30) and *mecA* (31) and two new assays specifically designed and validated for this study to detect *bla*_ACT_ and *qnrB*. The *bla*_ACT/MIR_ and *qnrB* assays detected multiple allelic variants of intended targets and did not detect *bla*_CMY_ or *qnrA1*/*qnrS1* sequences (Table S2). The *qnrB* assay did not detect *qnrB60* (cluster V), a genetically distant relative of the *qnrB1*/cluster I group targeted in this study. Accordingly, the prevalence of *qnrB* in NYC house mice may be higher than 7% (30/416) (Table 3). The *bla*_ACT/MIR_ resistance gene was detected most frequently (86/416, 21%). Four (1%) mice were positive for the methicillin resistance gene *mecA* (Table 3).

**TABLE 3:** PCR detection of AMR genes

AMR	Target gene	% prevalence by PCR at site:							% of positive mice by^a^:					Total
		M2	M3	Q1	X1	X2	X3	K1	Sex		Age			
									M	F	J	SA	A	
Methicillin resistance	*mecA*	1/42 (2.4)	2/119 (1.7)	1/210 (0.5)	0/38 (0.0)	0/1 (0.0)	0/1 (0.0)	0/5 (0.0)	3/4 (75)	1/4 (25)	1/4 (25)	0/4 (0)	3/4 (75)	4/416 (1.0)
Class A β-lactamase	*bla*_SHV_	1/42 (2.4)	0/119 (0.0)	7/210 (2.9)	1/38 (2.6)	0/1 (0.0)	0/1 (0.0)	0/5 (0.0)	4/8 (50)	4/8 (50)	0/14 (0)	2/14 (25)	6/14 (75)	8/416 (1.9)
Fluoroquinolone resistance	*qnrB*	6/42 (14.3)	10/119 (8.4)	12/210 (5.7)	2/38 (5.3)	0/1 (0.0)	0/1 (0.0)	0/5 (0.0)	23/30 (77)	7/30 (23)	7/30 (23)	5/30 (17)	18/30 (60)	30/416 (7.2)
Class C β-lactamase	*bla*_ACT/MIR_	14/42 (33.3)	19/119 (16.0)	47/210 (22.4)	6/38 (15.8)	0/1 (0.0)	0/1 (0.0)	0/5 (0.0)	58/86 (67)	28/86 (33)	26/86 (30)	14/86 (16)	46/86 (54)	86/416 (20.7)

a Abbreviations: A, adult; F, female; J, juvenile; M, male; SA, subadult.

### Distribution of AMR and bacterial genes.

To assess the observed diversity of bacterial and AMR genes in all individual mice (*n* = 416), we used results from the targeted PCR screening of anal swabs (or kidney for *Leptospira*) to assess whether carriage rates differed by site, sex, weight, or length. We found that 37% (153/416) of mice harbored at least one potentially pathogenic bacterium; 23% (96/416) were positive for at least one AMR gene (Table 4). One juvenile male mouse from M2 harbored five potentially pathogenic bacteria (C. difficile, C. perfringens, *Shigella*/EIEC, atypical EPEC, and K. pneumoniae); six mice each carried three AMR gene targets.

**TABLE 4:** Carriage of multiple bacteria and AMR determinants by individual mice following PCR screening of 12 bacterial and 4 AMR genes

Carriage type	No. (%) of mice with no. of detections:					
	0	1	2	3	4	5
Bacterial target	263 (63.2)	105 (25.2)	38 (9.1)	6 (1.4)	3 (0.7)	1 (0.2)
AMR gene	320 (76.9)	70 (16.8)	20 (4.8)	6 (1.4)	0 (0.0)	NA^a^

a NA, not applicable.

The highest average number of different pathogenic bacteria detected per mouse was at site M2 (0.74 per mouse). Fewer bacteria were detected per mouse in site X1 (0.21 per mouse) than any other site (excluding X2, X3, and K1, where sample size was fewer than five mice [Table 5]). The frequency of AMR genes mirrored the frequency of pathogenic bacteria: mice from site X1 (0.24 per mouse) had fewer AMR genes than those from M2, M3, and Q1 (Table 5). Pairwise comparisons of bacterial and AMR coinfection burden were made between all sites after adjusting for sex, length, and weight. Mice from X1 carried fewer pathogenic bacteria than did those from M2, M3, and Q1; however, when controlling the familywise error rate at the 0.05 level, the only statistically significant difference was found when comparing X1 to M2 (0.30-fold; 95% confidence interval [CI], 0.14, 0.65; *P* = 0.001) (Table 6). Pairwise comparisons also revealed that M3 mice carried fewer AMR genes than M2 (0.48-fold; 95% CI, 0.28, 0.82; *P* = 0.007) (Table 7). Weight and length were not associated with an increased coinfection burden, but we found that male mice carried more AMR genes than female mice (1.87-fold; 95% CI, 1.28, 2.73; *P* = 0.001) regardless of site, weight, or length.

**TABLE 5:** Average number of detections in individual mice at each collection site by PCR screening of 12 bacterial and 4 AMR genes

Type of detection	Avg no. of detections at site:						
	K1	M2	M3	Q1	X1	X2	X3
Bacterial target	0.00	0.74	0.50	0.56	0.21	0.00	0.00
AMR gene	0.00	0.52	0.26	0.31	0.24	0.00	0.00

**TABLE 6:** Pairwise comparison of bacterial coinfection load between sites

Site pairwise comparison	Fold change	95% confidence interval		*P* value
		Lower limit	Upper limit	
X1 vs M2	0.30	0.14	0.65	0.001^a^
X1 vs M3	0.40	0.19	0.85	0.01
X1 vs Q1	0.38	0.19	0.78	0.01
M2 vs M3	1.36	0.86	2.14	0.19
M2 vs Q1	1.28	0.85	1.92	0.23
M3 vs Q1	0.94	0.68	1.31	0.73

a Statistical significance controlling familywise error rate at 0.05 level. Sites K1, X2, and X3 were excluded as no AMR or bacterial genes were detected in mice from these sites.

**TABLE 7:** Pairwise comparison of AMR coinfection load between sites

Site pairwise comparison	Fold change	95% confidence interval		*P* value
		Lower limit	Upper limit	
X1 vs M2	0.46	0.21	1.01	0.05
X1 vs M3	0.96	0.45	2.06	0.92
X1 vs Q1	0.69	0.34	1.38	0.29
M2 vs M3	2.09	1.22	3.59	0.007^a^
M2 vs Q1	1.57	0.96	2.55	0.07
M3 vs Q1	0.75	0.48	1.18	0.21

a Statistical significance controlling familywise error rate at 0.05 level. Sites K1, X2, and X3 were excluded as no AMR or bacterial genes were detected in mice from these sites.

We also tested for associations between mouse characteristics (site of collection, sex, weight, and length) and the detection of individual bacterial or AMR genes. The likelihood of a mouse carrying *Shigella*/EIEC in M3 was higher than that for X1 (adjusted odds ratio [aOR], 26; 95% CI, 3.32, 3,330.02; *P* = 0.0002), although we failed to find significant differences in distributions between sites for any other bacterial agents (data not shown). Similarly, the likelihood of detection for the four AMR genes screened by PCR was not significantly associated with any collection site. Sex and mouse length were associated with an increased risk of detection for particular AMR genes. Males were more likely to be carrying *qnrB* or *bla*_ACT/MIR_. Longer mice were more likely to be positive for *bla*_SHV_ (Table 8).

**TABLE 8:** Association between mouse characteristics and prevalence of AMR genes

AMR gene	Mouse characteristics	aOR^a^	95% confidence interval		*P* value^b^
			Lower limit	Upper limit	
*mecA*	Male vs female	1.55	0.24	16.45	0.65
	Wt	1.32	0.82	2.06	0.24
	Length	0.24	0.02	2.71	0.24
*qnrB*	Male vs female	2.85	1.25	7.22	0.01*
	Wt	0.96	0.80	1.16	0.70
	Length	1.64	0.65	4.25	0.29
*bla*_SHV_	Male vs female	0.81	0.20	3.33	0.77
	Wt	0.72	0.48	1.02	0.06
	Length	8.70	1.60	54.65	0.01*
*bla*_ACT/MIR_	Male vs female	1.93	1.16	3.25	0.01*
	Wt	1.11	0.99	1.25	0.07
	Length	0.77	0.43	1.36	0.37

a Adjusted odds ratio (aOR) was calculated using Firth logistic regression analysis.

b Statistically significant associations are marked with an asterisk (familywise error rate controlled at 0.05 level).

### Persistence of bacteria and AMR genes.

**FIG 5:**
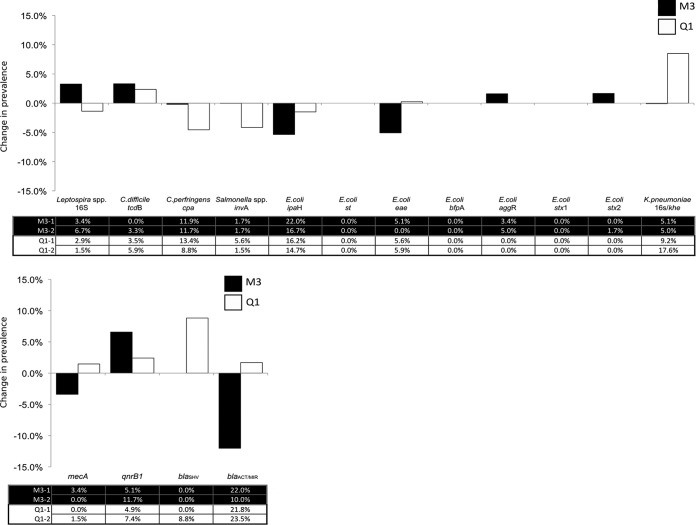
Change in PCR prevalence of bacterial (A) and antimicrobial resistance (B) genes between two time points at two sites in New York City.

Mice were trapped at two time points, 6 and 11 months apart at M3 and Q1 sites, respectively, to assess whether the carriage of bacteria or AMR genes persisted over an extended period at the community level. The most substantial changes were an 8.5% increase of K. pneumoniae carriage at Q1 and a 5.3% decrease of *Shigella*/EIEC at M3 (Fig. 5A, “E. coli *ipaH*”).

At site Q1, AMR gene carriage was lower at the first time point than the second (0.58-fold; 95% CI, 0.35, 0.95; *P* = 0.029), with all four AMR targets showing an increase in prevalence over the 11-month period (Fig. 5B). The prevalence of *bla*_SHV_ rose from 0.0% to 8.8% (aOR, 30.89; 95% CI, 3.47 to 4,068.02; *P* = 0.001).

## DISCUSSION

Recent studies of rats in North American cities have reported the presence of pathogenic bacteria (32–34). House mice occupy a niche position within many urban structures (e.g., homes, restaurants, and schools) wherein they have even more intimate contact with humans. Accordingly, we extended our NYC rodent surveillance analyses to mice. We sought to collect an even representation of animals from each borough. Access to certain sites and various degrees of infestation resulted in a higher representation of mice from one site in Manhattan (M3) and one in Queens (Q1).

Our 16S rRNA analyses of NYC mice reported here are most consistent with those obtained with wild house mice in rural Germany, where *Bacteroidetes* were the most dominant phylum, followed by *Firmicutes* and *Proteobacteria* (35). Other studies of house mouse feces from Germany, the United Kingdom, and the United States have identified *Firmicutes* or *Proteobacteria* as the most dominant populations of bacteria (36–38). In our study, *Proteobacteria* varied between sites, with average proportions ranging from 2.1% to 18%; these proportions are consistently higher than the 1% observed in mice from rural and urban areas in the United Kingdom (37) but lower than those of mice from rural Maryland and the District of Columbia, USA (26.1 to 36.7%) (38). Such variation between mouse populations may be associated with multiple factors, including diet, host genetics, and geography. 16S rRNA primer selection can also lead to biases in output data (39). With the exception of the analysis by Rosshart et al. (38), which also utilized the V4 region of the 16S rRNA gene, all other studies targeted different regions.

The microbial population structure of NYC house mice was consistent at two time points at two sites: M3, first sampling and 6 months later, and Q1, first sampling and 11 months later. This observation differs from findings obtained by Maurice et al. wherein wild wood mice sampled in their natural woodland habitat had dramatic seasonal changes in their gut microbiota (40). We speculate that the consistency of the NYC house mouse microbiome reflects consistency in their diet. Whereas wild wood mice experience profound seasonal changes in diet, house mice live in an environment where warmth and food are abundant year-round. Thus, they are not subject to the selective pressure that is characteristic of wild mice living outdoors.

Targeted screening for specific pathogenic bacteria by PCR identified a wide distribution of bacteria capable of causing gastrointestinal disease. These included *Shigella*/EIEC, atypical EPEC, STEC, C. difficile, and C. perfringens. We also found molecular evidence of Salmonella enterica, the leading cause of bacterial food poisoning outbreaks in the United States in which a single bacterial cause was identified (41). S. enterica causes a range of disease syndromes; however, it is most commonly associated with typhoid or enterocolitis and diarrhea (42). In the United States, there are approximately 1.4 million reported cases per year, causing 15,000 hospitalizations and 400 deaths (43). *Salmonella* is transmitted by the fecal-oral route, mostly from food contaminated with animal feces (44), and it has been isolated from house mice in urban and rural areas (8, 45). We cannot discern from current data whether mice contribute to human *Salmonella* infections.

Pathogenic species of *Leptospira* can cause pulmonary hemorrhage syndrome, undifferentiated fever, and Weil’s disease (reviewed in reference 46). Cases are rarely reported in NYC, although a recent cluster of three cases in the Bronx resulted in one death (47), highlighting the potential health threat presented by this zoonotic agent. We identified two distinct genotypes of *Leptospira*, one that was distributed across Queens, Manhattan, and the Bronx (group 1) and a second that was confined to Manhattan (group 2). We considered the possibility that ecological factors might contribute to differences in genotype distribution. The built environments of the Manhattan trapping locations were similar to those in Queens and the Bronx where *Leptospira* was detected—subbasement compactor rooms (where waste is contained) with direct access to the external environment. Perhaps as-yet-undetermined physical barriers limit the movement of mice, and thus *Leptospira*, between sites. Our findings suggest that although rats are commonly associated with dispersal of *Leptospira* (32, 33), surveillance for potentially pathogenic *Leptospira* should be extended to house mice.

The resistome (total pool of AMR in the environment) is influenced by pressure from anthropological factors, including human medicine and agricultural use of antibiotics as growth promoters, that can lead to evolutionary selection of resistant bacteria (48). Urban rodents have been linked to carriage of antibiotic-resistant bacteria, including E. coli (17) and Staphylococcus aureus (16). Even wild, presumably antibiotic-naive populations of rodents (bank voles and wood mice) have been found to carry resistant *Enterobacteriaceae* (15). We found that house mice were carrying a diverse array of AMR genes. There was an increase in carriage of all screened AMR genes (*mecA*, *qnrB*, *bla*_SHV_, and *bla*_ACT/MIR_) in samples longitudinally collected from Fresh Meadows (Q1). A similar upward trend was observed in Chelsea (M3) for one gene (*qnrB*), but there was a decrease in carriage of *bla*_ACT/MIR_ and *mecA*. *bla*_SHV_, a β-lactamase responsible for broad-spectrum resistance to penicillin (49), was most noteworthy as it dramatically increased in prevalence over the 11-month period between mouse collections at site Q1. We have no explanation for this increase in prevalence; it may represent normal variation or as-yet-undetermined selection factors. We also found that male mice had an increased number of AMR genes compared to females. This may reflect the expanded home range of male mice (50), resulting in increased events of exposure to AMR-positive bacteria.

The epidemiology of community-acquired C. difficile infection (CA-CDI), a major burden on the health care system, is not well understood (51). The majority of CDI cases are believed to cycle directly between hospitals and the community; in one study, 79% of isolates from health care-associated (HA) CDI cases matched those from the community (52). The maintenance and amplification of C. difficile by reservoirs in the urban environment may contribute to this cycle. Recently, wild urban Norway and black rats in Vancouver were found to harbor C. difficile and to shed specific ribotypes associated with CDI (34). We reported elsewhere that 0.8% of Norway rats in NYC were positive for C. difficile DNA (32); this is a lower prevalence rate than the 4.3% rate that we report here in house mice. To our knowledge, this is the first report documenting the detection and isolation of C. difficile from wild house mice in an urban setting. Importantly, we detected ribotypes previously associated with CA-CDI (53). One of these ribotypes, RT106, was the most common cause of CA-CDI identified in a 10-site population-based human surveillance study in 2014 (53). Although these results do not prove that house mice contribute to the transmission cycle of CA-CDI in human populations in NYC, they are sufficiently intriguing to merit testing for potential links of human cases to local infestation with C. difficile-infected mice.

## MATERIALS AND METHODS

### Mouse collection.

A total of 416 mice comprised of 221 males (107 adults, 29 subadults, and 85 juveniles) and 195 females (93 adults, 30 subadults, and 72 juveniles) were collected from seven sites in NYC (Fig. 1). The majority (410 mice) were trapped in or around the building trash compactor rooms located in the subbasement of multifamily residential buildings at five locations (M2, M3, Q1, X1, and X2). The remaining mice were caught in a commercial building (kitchen and food storage area) (K1, *n* = 5) or a private single-family residence (X3, *n* = 1).

Mice were live-trapped using single (SFA folding trap; Sherman, Tallahassee, FL) and multiple (Pro-Ketch [Kness, Albia, IA] and Tin Cat [Victor, Lititz, PA]) live catch traps from four boroughs in NYC: Manhattan, Queens, Brooklyn, and the Bronx. For sites M3 and Q1, a second site visit occurred 6 and 11 months after the first trapping, respectively (designated site-1 and site-2). Following euthanasia by exposure to a lethal dose of CO_2_ per the American Veterinary Medical Association guidelines, mice were weighed, sexed, and measured for length (an indirect measure for age) from the tip of the nose to the base of the tail. Anal swab and tissue samples from each mouse were collected and snap-frozen on dry ice. Fecal pellets were also removed from traps, and if traps contained multiple mice, tubes were labeled as “pooled fecal pellets.” Thirty-seven mice from Q1-1 and one mouse from Q1-2 were swabbed; however, no organs were collected. Procedures described here were approved by the Institutional Animal Care and Use Committee at Columbia University (protocol number AC-AAAE8351/AC-AAAE8450).

### 16S rRNA library preparation.

Nucleic acid for 16S rRNA analyses and AMR screening was extracted from fetal pellet samples (either pooled or individual; *n* = 131) using the Fast DNA stool minikit (Qiagen) with the following modifications. Homogenized fetal pellets in phosphate-buffered saline were added to UV-irradiated 2-ml tubes containing 0.1-mm and 0.5-mm glass beads (Mo Bio Laboratories, Carlsbad, CA) and 1 ml InhibitEX buffer and vortexed for 1 min. Negative controls containing reagents only were included for every 10 to 12 samples. Tubes were heated for 5 min at 70°C and placed on the TissueLyser II (Qiagen) at maximum speed (30 kHz) for 5 min. Samples were heated a second time at 70°C for a further 5 min before centrifugation at maximum speed for 5 min. Six hundred microliters of supernatant was added to a UV-irradiated 2-ml tube containing 25 µl proteinase K (Qiagen) and vortexed before the addition of prewarmed AL buffer. The remainder of the extraction protocol followed kit instructions. Nucleic acid was eluted in 50 µl ATE buffer, and concentration and purity were determined using the NanoDrop-1000 spectrophotometer (Thermo Fisher Scientific, Waltham, MA).

Due to constraints on the number of available unique barcodes, 110 fecal pellet samples were selected representing each site and time point for 16S rRNA analysis. PCR master mix was prepared by adding 11.35 µl DNA-free PCR water (Qiagen), 2 µl PCR buffer II (Thermo Fisher Scientific), 0.15 µl (0.75 U) of AccuPrime *Taq* DNA polymerase (Thermo Fisher Scientific), and 0.5 µl Sau3AI (2.5 U) (New England Biolabs, Ipswich, MA) and then UV irradiated and aliquoted into 96-well PCR plates. Primers that include unique 12-nt barcode sequences (54) were added to each well prior to incubation at 37°C for 30 min to allow Sau3AI to digest contaminating double-stranded DNA (dsDNA). Plates were chilled prior to the addition of 50 ng sample DNA and cycling using the following conditions: 94°C for 5 min; 35 cycles of 94°C for 20 s, 53°C for 25 s, and 68°C for 45 s; and 68°C for 10 min. PCR products were visualized by gel electrophoresis, purified using Agencourt AMPure XP beads (Beckman Coulter, Brea, CA), quantified via qPCR (Kapa library quantification kit; Kapa Biosystems, Boston, MA), pooled to equimolar concentrations, and run on the MiSeq system (Illumina, San Diego, CA) using MiSeq reagent kit V3 (600 cycle) with 15% PhiX spike-in.

### Bioinformatics.

16S rRNA PCR product sequencing was performed on the MiSeq platform and analyzed using FastQC v0.11.5 software (55). Regions with low complexity and low quality scores were trimmed using SeqTK v1.2 software (56). After trimming, 16S rRNA sequences were analyzed using the QIIME (v1.9) pipeline (57). Reads were demultiplexed and quality filtered using QIIME scripts, and open-reference operational taxonomic unit picking was performed. The percentage of reads mapping to each bacterial phylum was calculated for all samples. Samples were then grouped according to site or site/time point and average abundances, and an average abundance was generated.

### Antimicrobial resistance molecular testing.

DNA extracted from fecal pellet samples (*n* = 131) using the Fast DNA stool minikit (Qiagen) was divided into seven pools according to site and time point (M2, M3-1, M3-2, Q1-1, Q1-2, X1/2/3, and K1). Equimolar concentrations of DNA from each sample were combined to achieve a final total of 500 ng DNA for each pool. Each pool was aliquoted to a new plate according to kit instructions for the microbial DNA qPCR array (Qiagen). All plates, including a negative-control plate, were run on the CFX96 Touch real-time PCR detection system (Bio-Rad, Hercules, CA), and results were assessed using the data analysis software. *C*_*T*_ values of <30 were considered strongly positive, those of <34 were considered positive, those of 34 to 37 were considered inconclusive, and those of >37 were considered negative, per the manufacturer’s recommendations. The heat map was prepared in Microsoft Excel.

### Targeted PCR analyses.

Nucleic acid was extracted from kidney tissue (*n* = 378) using the AllPrep DNA/RNA minikit (Qiagen) and from anal swabs (*n* = 416) using the easyMAG automated platform (bioMérieux, Boxtel, The Netherlands). Nucleic acid concentration and purity were determined on the NanoDrop-1000 spectrophotometer (Thermo Scientific).

Based on bacterial and AMR results obtained from 16S rRNA and qPCR analysis, respectively, 11 bacterial and 4 AMR genes were selected for direct PCR screening of anal swabs. In addition, direct PCR for *Leptospira* spp. was performed on kidney DNA while all other PCRs were performed on total nucleic acid from anal swabs. Extracted nucleic acid was verified as inhibitor free by performing PCR for host targets glyceraldehyde-3-phosphate dehydrogenase (anal swab) or M. musculus mitochondrial D-loop (kidney). Extended 16S rRNA sequences for *Leptospira* spp. were obtained using a nested PCR targeting a 1,500-nt region. To ensure primer specificity, PCR products from all screening assays were sequenced to confirm identity. Primers, cycling conditions, and gene targets for all assays are detailed in Table S3 in the supplemental material.

10.1128/mBio.00624-18.3 TABLE S3  PCR primers and thermocycling conditions. Download TABLE S3, DOCX file, 0.2 MB. Copyright © 2018 Williams et al. 2018 Williams et al. This content is distributed under the terms of the Creative Commons Attribution 4.0 International license.

### Validation of newly designed PCR assays.

Primers used in these assays were assessed for potential *in silico* cross-reactivity using Primer-BLAST (https://www.ncbi.nlm.nih.gov/tools/primer-blast) with default settings against the nonredundant database and a maximum PCR product size of 300 nt (58). The three bacterial PCR assays designed for use in this study were tested against typed material (Table S2). PCR for the C. perfringens *alpha*-toxin (*cpa*) was assessed using two C. perfringens strains as well as C. difficile and Clostridium tetani to test for cross-reactivity. Two PCR assays for diarrheagenic E. coli were also developed for this study. PCR for typical EPEC (*bfpA*) was assessed against a panel of EPEC strains possessing a diverse range of bundle-forming pilus sequences, including representatives of each clonal group (alpha, *n* = 3; beta, *n* = 2). Related *Enterobacteriaceae* from the genus *Shigella* were included to test specificity. The presence of *ipaH*, a multicopy chromosomal and plasmid gene, uniquely defines EIEC and *Shigella* spp. (59–61). PCR for this gene was tested against two Shigella flexneri strains and Shigella sonnei, as well as six non-EIEC strains to test for cross-reactivity.

Two AMR PCR assays were also developed for use in this study. *bla*_ACT/MIR_ was tested against three bacterial strains containing different ACT alleles and one strain possessing the MIR allele (high nucleotide identity with ACT) (62) (Table S2). To test for nonspecific amplification, the assay was also screened against an isolate possessing a different AmpC β-lactamase, *bla*_CMY_. PCR primers targeting the *qnrB* gene were also assessed (Table S2). The *qnrB* AMR family is highly diverse and separated into seven phylogenetically defined clusters (63). PCR primers used in this study were designed to confirm *qnrB* cluster I (defined as QnrB-1 group in the qPCR array; *qnrB*-1, -2, -3, -6, -7, -9, -13, -14, -15, -16, -17, -18, -20, -23, -24, -29, and -30); however, due to sequence similarity across the primer binding regions, detection of all other clusters, excluding clusters IV and V, is possible. Typed strains representing three clusters (I, III, and V) were included in preliminary validation studies to test for specificity. Related *qnr* AMR genes, A and S, were included to test for cross-reactivity. PCR products were sequenced to confirm identity.

### Phylogenetics.

16S rRNA gene sequences obtained from 12 samples, as well as the positive control used during PCR screening, were compared with available *Leptospira* sequences representing pathogenic, intermediate, and saprophytic strains. Nucleotide alignment was performed in Geneious 10.1.2 (64) and exported to MEGA7 (65). A maximum likelihood tree was prepared with the Kimura two-parameter model (K2) using discrete gamma distribution (+G) with invariant sites (+I) and 500 bootstraps. Newick trees were exported to FigTree (http://tree.bio.ed.ac.uk/software/figtree/) for annotation. The final tree displays bootstrap support values when above 70%.

### Culture and molecular characterization of Clostridium difficile*.*

C. difficile culture was attempted on select fecal pellet samples sourced from mice where cytotoxin B (*tcdB*) DNA was detected by PCR in the anal swab. At site Q1, *tcdB*^+^ anal swab samples were from mice where only pooled fecal pellets (i.e., pellets from ≥2 mice) were available. Fifty microliters of the fecal suspension was spread onto prereduced ChromID C. difficile agar plates (bioMérieux), incubated at 37°C under anaerobic conditions, and assessed for typical C. difficile colonies after 48 h. Presumptive colonies were simultaneously subcultured onto prereduced blood agar and inoculated into PCR mix to test for the presence of *tcdB*. Subcultures that displayed typical colony morphology on blood agar and were *tcdB* DNA positive by PCR were then inoculated into 6 ml prereduced reinforced clostridial medium (Becton, Dickinson, Sparks, MD) for overnight growth and subsequent storage. Stored isolates were regrown and tested against a multiplex real-time PCR assay that includes cytotoxin A and B (*tcdA* and *tcdB*) and binary toxin (*cdtA* and *cdtB*) genes (Table S3). The presence of any mutations within the *tcdC* gene was assessed by PCR and fragment analysis. Ribotyping of C. difficile isolates was performed by capillary gel electrophoresis as previously described (66).

### Statistical analyses.

Data were analyzed using Matlab and Statistics Toolbox release 2013a (The MathWorks, Natick, MA). Multiple comparisons were corrected using Hochberg’s step-up procedure (67) controlling the familywise error rate at a level of α = 0.05. All reported *P* values were two-tailed.

For each bacterial species or AMR gene detected, we tested the association between its presence and site or demographic variables by fitting a logistic regression model using the binary agent presence (versus absence) status as the dependent variable and using site, length, weight, and sex as independent variables. Because not all agents were found at all sites, we applied Firth logistic regression (68) to deal with the quasicomplete separation phenomenon. Adjustments were made for multiple comparisons within bacteria and AMR genes separately (12 bacteria or 4 AMR genes, multiplied by 10 pairwise site comparisons).

In M3 and Q1, where serial collections were obtained, we again applied the Firth logistic regression to test for association between time point and the prevalence of individual bacterial or AMR genes detected in that site, adjusting for sex, weight, and length.

We also tested the association between agent richness and site or demographic variables. The count of bacterial species (or AMR genes) was fitted into a Poisson regression model as the dependent variable. Site, length, weight, and sex were used as independent variables. The familywise error rate was controlled at the 0.05 level for the 10 pairwise site comparisons.

### Accession number(s).

Nucleotide sequences of leptospiral extended 16S rRNA are deposited in GenBank (accession numbers MF497795 to MF497806). Raw Illumina 16S rRNA files have been deposited in the SRA database under GenBank accession number SRP136544.
