# Supplementary material for: New York City House Mice (Mus musculus) as Potential Reservoirs for Pathogenic Bacteria and Antimicrobial Resistance Determinants
Source: mBio. 2018 Apr 17;9(2):e00624-18. doi: 10.1128/mBio.00624-18 (PMC5904414; doi:10.1128/mBio.00624-18)
Supplement: TABLE S1 [file mbo002183843st1.docx]

| Phylum | M2 | M3 | Q1 | X1 | X2 | X3 | K1 |
| --- | --- | --- | --- | --- | --- | --- | --- |
| Unassigned | 0.09% | 0.15% | 0.11% | 0.08% | 0.04% | 0.23% | 0.14% |
| Bacteriodetes | 59.50% | 42.49% | 48.25% | 45.13% | 75.87% | 47.61% | 43.95% |
| Deferribacteres | 0.13% | 0.40% | 0.09% | 0.13% | 0.05% | 1.73% | 1.60% |
| Firmicutes | 29.97% | 35.88% | 35.22% | 34.30% | 21.75% | 37.42% | 35.28% |
| Fusobacteria | 2.44% | 0.57% | 0.17% | 0.01% | 0.00% | 0.00% | 0.00% |
| Proteobacteria | 5.89% | 18.23% | 12.97% | 15.40% | 2.12% | 12.00% | 17.58% |
| Spirochetes | 0.01% | 0.05% | 0.00% | 0.00% | 0.00% | 0.00% | 0.00% |
| Tenericutes | 0.23% | 0.37% | 0.47% | 0.51% | 0.15% | 0.99% | 0.18% |
| Verrucomicrobia | 1.74% | 1.87% | 2.71% | 4.43% | 0.02% | 0.00% | 1.26% |

**Table S1A.** Microbiome composition determined from 16S V4 sequencing of pooled fecal pellets from NYC house mice at seven unique sites. Values represent average proportions of operational taxonomic units assigned to each phylum

**Table S1B.** Microbiome composition determined from 16S V4 sequencing of pooled fecal pellets from NYC house mice at two sites, each with two collection time points. Values represent average proportions of operational taxonomic units assigned to each phylum

| Phylum | M3-1 | M3-2 | Q1-1 | Q1-2 |
| --- | --- | --- | --- | --- |
| Date of collection | 09-10/2014 | 03-04/2015 | 10-11/2014 | 09/2015 |
| Unassigned | 0.10% | 0.20% | 0.10% | 0.12% |
| Bacteriodetes | 39.63% | 44.83% | 51.30% | 44.07% |
| Deferribacteres | 0.34% | 0.45% | 0.06% | 0.14% |
| Firmicutes | 30.92% | 39.95% | 34.31% | 36.46% |
| Fusobacteria | 0.01% | 1.03% | 0.26% | 0.04% |
| Proteobacteria | 26.29% | 11.61% | 12.04% | 14.26% |
| Spirochetes | 0.00% | 0.08% | 0.00% | 0.00% |
| Tenericutes | 0.37% | 0.36% | 0.29% | 0.72% |
| Verrucomicrobia | 2.34% | 1.49% | 1.64% | 4.18% |
