# Supplementary material for: New York City House Mice (Mus musculus) as Potential Reservoirs for Pathogenic Bacteria and Antimicrobial Resistance Determinants
Source: mBio. 2018 Apr 17;9(2):e00624-18. doi: 10.1128/mBio.00624-18 (PMC5904414; doi:10.1128/mBio.00624-18)
Supplement: TABLE S2 [file mbo002183843st2.docx]

**Supplemental Table 2.** Typed strains used for PCR assay specificity testing

| Target gene | Organism | ID | Strain information | Variant | Source | PCR result |
| --- | --- | --- | --- | --- | --- | --- |
| *cpa* | *Clostridium perfringens* | ATCC 13124 ^TM^ | NCTC8237 |  | ATCC | + |
|  | *Clostridium perfringens* | HM-310 | WAL-14572 |  | BEI | + |
|  | *Clostridium tetani* | ATCC 19406 ^TM^ | Flugge |  | ATCC | - |
|  | *Clostridium difficile* | ATCC 6989 ^TM^ | Hall and O’Toole Prevot |  | ATCC | - |
|  |  |  |  |  |  |  |
| *bfpA* | *Shigella flexneri* | #423 |  |  | FDA-CDC | - |
|  | *Shigella flexneri* | NR-517 | Serotype 2A 24570 |  | BEI | - |
|  | *Shigella sonnei* | NR-519 | WRAIR I virulent |  | BEI | - |
|  | *Escherichia coli* | TW07897 | C771 | *bfp*A b-5 | STEC | + |
|  | *Escherichia coli* | TW07923 | RN587/1 | *bfp*A b-6 | STEC | + |
|  | *Escherichia coli* | TW08262 | RN410/1 | *bfp*A a-3 | STEC | + |
|  | *Escherichia coli* | TW08261 | RN191/1 | *bfp*A a-2 | STEC | + |
|  | *Escherichia coli* | TW07887 | E990 | *bfp*A a-1 | STEC | + |
|  | *Escherichia coli* | NR-9296 | B171 | *bfp*A a-2 | BEI | + |
|  |  |  |  |  |  |  |
| *ipaH* | *Shigella flexneri* | #0423 |  |  | FDA-CDC | + |
|  | *Shigella flexneri* | NR-517 | Serotype 2A 24570 |  | BEI | + |
|  | *Shigella sonnei* | NR-519 | WRAIR I virulent |  | BEI | + |
|  | *Escherichia coli* | TW07897 | C771 |  | STEC | - |
|  | *Escherichia coli* | TW07923 | RN587/1 |  | STEC | - |
|  | *Escherichia coli* | TW08262 | RN410/1 |  | STEC | - |
|  | *Escherichia coli* | TW08261 | RN191/1 |  | STEC | - |
|  | *Escherichia coli* | TW07887 | E990 |  | STEC | - |
|  | *Escherichia coli* | NR-9296 | B171 |  | BEI | - |
|  |  |  |  |  |  |  |
| *bla*_ACT/MIR_ | *Enterobacter cloacae* complex | NR0242 |  | ACT-5 | DoID | + |
|  | *Enterobacter cloacae* | #0163 |  | ACT-7 | FDA-CDC | + |
|  | *Enterobacter cloacae* | NR0248 |  | ACT-16 | DoID | + |
|  | *Enterobacter cloacae* | NR2283 |  | MIR-3 | DoID | + |
|  | *Citrobacter freundii* | NR0091 |  | CMY | DoID | - |
|  |  |  |  |  |  |  |
| *qnrB* | *Citrobacter braakii* | ATCC 51113 ^TM^ | Brenner et al. | *qnr*B61  cluster III | ATCC | + |
|  | *Citrobacter freundii* | ATCC 6879^TM^ | Werkman and Gillen | *qnr*B60  cluster V | ATCC | - |
|  | Salmonella Corvallis | #0406 |  | *qnr*S1 | FDA-CDC | - |
|  | Salmonella Concord | #0407 |  | *qnr*A1 | FDA-CDC | - |
|  | *Shigella flexneri* | #0423 |  | *qnr*B6  cluster I | FDA-CDC | + |

DoID, Department of Infectious Diseases, Columbia University; FDA CDC, Food and Drug Administration-Centers for Disease Control and Prevention Antimicrobial Resistance Isolate Bank; ATCC, American Type Culture Collection; BEI, Biodefence and Emerging Infectious Research Resources Repository; STEC, STEC Center, Department of Microbiology and Molecular Genetics, Michigan State University; *bfp*A x-y, bundle-forming pili A classification; ACT, AmpC-type β-lactamase.
