# Supplementary material for: New York City House Mice (Mus musculus) as Potential Reservoirs for Pathogenic Bacteria and Antimicrobial Resistance Determinants
Source: mBio. 2018 Apr 17;9(2):e00624-18. doi: 10.1128/mBio.00624-18 (PMC5904414; doi:10.1128/mBio.00624-18)
Supplement: TABLE S3 [file mbo002183843st3.docx]

**Supplemental Table 3. PCR primers and thermocycling conditions**

| **House keeping genes** |  | Forward primer (5’-3’) | Reverse primer (5’-3’) | Product size (nt) | AT (°C) | Reference |
| --- | --- | --- | --- | --- | --- | --- |
| Glyceraldehyde 3-phosphate dehydrogenase |  | TGACGTGCCGCCTGGAGAAA | AGTGTAGCCCAAGATGCCCTTCAG | 98 | 60* | (1) |
| *M. musculus* mitochondrial d-loop |  | TGAATTGGAGGACAACCAGT | TTATAAGGCCAGGACCAAAC | 1240 | 58-53& | (2) |

| **Bacterial screening PCR** | Target gene | Forward primer (5’-3’) | Reverse primer (5’-3’) | Product size (nt) | AT (°C) | Reference |
| --- | --- | --- | --- | --- | --- | --- |
| *C. difficile* | *tcd*B | GAAAGTCCAAGTTTACGCTCAAT | GCTGCACCTAAACTTACACCA | 177 | 55 | (3) |
| *C. perfringens* | *cpa* | CGTTGATAGCGCAGGACATG | TACCTTTGCTGCATAATCCCAAT | 244 | 56 | This study |
| *Salmonella* | *inv*A | TCGTCATTCCATTACCTACC | AAACGTTGAAAAACTGAGGA | 119 | 54 | (4) |
| Enteroinvasive *E. coli / Shigella* | *ipa*H | ATTGCCCGGGATAAAGTCAGA | GCAGTGGAGAGCTGAAGTTTCTC | 101 | 55 | This study |
| Enterotoxigenic *E. coli* | *St* | ATTTTTMTTTCTGTATTRTCTT | CACCCGGTACARGCAGGATT | 190 | 50 | (5) |
| Enteropathogenic *E. coli* – atypical/typical | *eae* | CATTGATCAGGATTTTTCTGGTGATA | CTCATGCGGAAATAGCCGTTA | 102 | 55 | (6) |
| Enteropathogenic *E. coli* – typical | *bfp*A | TTGGATAAAGCGGCATGTGTT | CGCTTCAGCAGGAGTAATAGCA | 109 | 55 | This study |
| Enteroaggregative *E. coli* | *agg*R | CAGCGATACATTAAGACGCCTAAAG | CGTCAGCATCAGCTACAATTATTCC | 116 | 55 | (7) |
| Shiga toxin-producing *E. coli* | *stx*1 | GCGTTCTTATGTAATGACTGCTGAAG | AGAAATTCTTCCTACACGAACAGAGTC | 118 | 60 | (8) |
| Shiga toxin-producing *E. coli* | *stx*2 | TGCATCCAGAGCAGTTCTGC | CGGCGTCATCGTATACACAGG | 121 | 60 | (8) |
| *K. pneumoniae* – screen | 16S | ATTTGAAGAGGTTGCAAACGAT | TTCACTCTGAAGTTTTCTTGTGTTC | 130 | 61 | (9) |
| *K. pneumoniae* – confirmatory | *khe* | TGATTGCATTCGCCACTGG | GGTCAACCCAACGATCCTG | 428 | 60 | (10) |
| *Leptospira* spp. – screen | 16S-23S ITS | GGCGGCGCGTCTTAAACATG | TTCCCCCCATTGAGCAAGATT | 330 | 63 | (11) |
| *Leptospira* spp*. –* supplemental R1 | 16S rRNA | AGAGTTTGATCCTGGCTCAG | AAGGAGGTGATCCAGCC | 1500 | 42 | (12) |
| *–* supplemental R2 | 16S rRNA | GGCGGCGCGTCTTAAACATGC | TGTGTACAAGGTCCGGGAAC | 1328 | 64 | (13) |

| **Antimicrobial resistance target** | Target gene | Forward primer (5’-3’) | Reverse primer (5’-3’) | Product size (nt) | AT (°C) | Reference |
| --- | --- | --- | --- | --- | --- | --- |
| Methicillin resistance | *mec*A | CCTTGTCCGTAACCTGAATCAGCT | GCTAGAGTAGCACTCGAATTAGGCAGT | 164 | 60 | (14) |
| Fluroquinolone resistance | *qnr*B | CGCGACGTTCAGTGGTTC | GCAACGAYGCCTGGTAGTTG | 167 | 57 | This study |
| Class A β-Lactamase^ | *bla*_SHV_ | TTATCTC**Y**CTGTTAGCC**V**CC | TTTCGCTGACCGGCGWGTAG | 297 | 57 | (15) |
| Class C β-Lactamase | bla_ACT/MIR_ | TACAGGTACCGGATGAGGTC | GTATGGTCCAGCTTGAGCG | 205 | 57 | This study |

| ***C. difficile* characterization** | Target gene | Forward primer (5’-3’) | Reverse primer (5’-3’) | Product size (nt) | AT (°C) | Reference |
| --- | --- | --- | --- | --- | --- | --- |
| Multiplex PCR | Non-toxigenic$ | AGTATCATTTCACGAAGAGGAGCTAACAG | TCTGCTAATAGCAAATTATTTTAAATTAAATCTAC | 114 | 60 | (16) |
|  | Probe: | Q670-AGATAAAGTCGTTAGAAAACTG-BHQ3 |  |  |  |  |
|  | *tcd*A | ATTTTGGCGGAGTATATTTAGATGTTG | GTCTAGTCCAATAGAGCTAGGTCT | 95 | 60 |  |
|  | Probe: | Quas570-TTCCAGGTATTCACTCTGA-BHQ2 |  |  |  |  |
|  | *tcd*B | GATGCRGCYAAAGTTGTTGAATTAG | ATTAYAGGTAATCCTTCAGATAATGTAGGA | 89 | 60 |  |
|  | Probe: | CalRd610-CAACTGCAYTAGATGAAACT-BHQ2 |  |  |  |  |
|  | *cdt*A | ACTGTATATAGAAGATCTGGTCCTCAAGAA | GYTTGTCCTTCCCATTTTGATT | 116 | 60 |  |
|  | Probe: | Hex-TTTGGTTTAACYCTTACTTCC-BHQ1 |  |  |  |  |
|  | *cdt*B | GATCCGTTAGTTGCAGCRTATCC | TGATCAGTAGAGGCATGTTCATTTG | 86 | 60 |  |
|  | Probe: | Fam-ATTGTTGGAGTAGGTATGGAA-BHQ1 |  |  |  |  |
| HRCE | *tcd*C deletion | Hex-GCACCTCATCACCATCTTC | TGGTTCAAAATGAAAGACGAC | 249 | 53+ | (17, 18) |

AT, annealing temperature; *assay performed as a SYBR-green real-time PCR with 60s combined anneal and extension; &touchdown PCR (delta 0.5°C/cycle for 10 cycles); ^Forward primer modified as shown in bold text, reverse primer designed in this study; $PCR screen for non-toxigenic clostridial species; R1, PCR round 1; R2, PCR round 2; +assay performed as high-resolution capillary electrophoresis (HRCE)

**References**

1. Mamo S, Gal AB, Bodo S, Dinnyes A. 2007. Quantitative evaluation and selection of reference genes in mouse oocytes and embryos cultured in vivo and in vitro. BMC Dev Biol 7:14.

2. Gabriel SI, Stevens MI, Mathias Mda L, Searle JB. 2011. Of mice and 'convicts': origin of the Australian house mouse, *Mus musculus*. PLoS One 6:e28622.

3. van den Berg R, van Coppenraet L, Gerritsen H-J, Endtz H, van der Vorm E, Kuijper EJ. 2005. Prospective multicenter evaluation of a new immunoassay and real-time PCR for rapid diagnosis of *Clostridium difficile*-associated diarrhea in hospitalised patients. J Clin Microbiol 43:5338-5340.

4. Hoorfar J, Ahrens P, Radstrom P. 2000. Automated 5' nuclease PCR assay for identification of *Salmonella enterica*. J Clin Microbiol 38:3429-35.

5. Stacy-Phipps S, Mecca JJ, Weiss JB. 1995. Multiplex PCR assay and simple preparation method for stool specimens detect enterotoxigenic *Escherichia coli* DNA during course of infection. J Clin Microbiol 33:1054-9.

6. Nielsen EM, Andersen MT. 2003. Detection and characterization of verocytotoxin-producing *Escherichia coli* by automated 5' nuclease PCR assay. J Clin Microbiol 41:2884-93.

7. Hidaka A, Hokyo T, Arikawa K, Fujihara S, Ogasawara J, Hase A, Hara-Kudo Y, Nishikawa Y. 2009. Multiplex real-time PCR for exhaustive detection of diarrhoeagenic *Escherichia coli*. J Appl Microbiol 106:410-20.

8. Antikainen J, Kantele A, Pakkanen SH, Laaveri T, Riutta J, Vaara M, Kirveskari J. 2013. A quantitative polymerase chain reaction assay for rapid detection of 9 pathogens directly from stools of travelers with diarrhea. Clin Gastroenterol Hepatol 11:1300-1307 e3.

9. Liu Y, Liu C, Zheng W, Zhang X, Yu J, Gao Q, Hou Y, Huang X. 2008. PCR detection of *Klebsiella pneumoniae* in infant formula based on 16S-23S internal transcribed spacer. Int J Food Microbiol 125:230-5.

10. Chen Z, Liu M, Cui Y, Wang L, Zhang Y, Qiu J, Yang R, Liu C, Zhou D. 2014. A novel PCR-based genotyping scheme for clinical *Klebsiella pneumoniae*. Future Microbiol 9:21-32.

11. Merien F, Amouriaux P, Perolat P, Baranton G, Saint Girons I. 1992. Polymerase Chain Reaction for Detection of *Leptospira* spp. in Clinical Samples. Journal of Clinical Microbiology 30:2219-2224.

12. Weisburg W, Barns S, Pelletier D, Lane D. 1991. 16S ribosomal DNA amplification for phylogenetic study. Journal of Bacteriology 173:697-703.

13. Matthias MA, Diaz MM, K. C, M. C, Willig MR, V. P, E. G, R. G, Vinetz JM. 2005. Diversity of bat-associated *Leptospira* in the Peruvian amazon inferred by Bayesian phylogenetic analysis of 16S ribosomal DNA sequences. Am J Trop Med Hyg 73:964-974.

14. Gordon CL, Tokarz R, Briese T, Lipkin WI, Jain K, Whittier S, Shah J, Connolly ES, Yin MT. 2015. Evaluation of a multiplex polymerase chain reaction for early diagnosis of ventriculostomy-related infections. J Neurosurg 123:1586-92.

15. Weill FX, Demartin M, Tande D, Espie E, Rakotoarivony I, Grimont PA. 2004. SHV-12-like extended-spectrum-beta-lactamase-producing strains of *Salmonella enterica* serotypes Babelsberg and Enteritidis isolated in France among infants adopted from Mali. J Clin Microbiol 42:2432-7.

16. Avillan J, Granade M, Kitchel B, Karlsson M, Limbago B. 2015. Multiplex Real-time PCR for Toxin Profiling of *Clostridium difficile,* abstr Interscience Conference on Antimicrobial Agents and Chemotherapy, San Diego, September 19. American Society for Microbiology.

17. Killgore G, Thompson A, Johnson S, Brazier J, Kuijper E, Pepin J, Frost EH, Savelkoul P, Nicholson B, van den Berg RJ, Kato H, Sambol SP, Zukowski W, Woods C, Limbago B, Gerding DN, McDonald LC. 2008. Comparison of seven techniques for typing international epidemic strains of *Clostridium difficile*: restriction endonuclease analysis, pulsed-field gel electrophoresis, PCR-ribotyping, multilocus sequence typing, multilocus variable-number tandem-repeat analysis, amplified fragment length polymorphism, and surface layer protein A gene sequence typing. J Clin Microbiol 46:431-7.

18. Abrahamian FM, Talan DA, Krishnadasan A, Citron DM, Paulick AL, Anderson LJ, Goldstein EJC, Moran GJ, Group EMINS. 2017. *Clostridium difficile* Infection Among US Emergency Department Patients With Diarrhea and No Vomiting. Ann Emerg Med 70:19-27 e4.
